# Natural canopy bridges effectively mitigate tropical forest fragmentation for arboreal mammals

**DOI:** 10.1038/s41598-017-04112-x

**Published:** 2017-06-20

**Authors:** Tremaine Gregory, Farah Carrasco-Rueda, Alfonso Alonso, Joseph Kolowski, Jessica L. Deichmann

**Affiliations:** 1 Center for Conservation and Sustainability, Smithsonian Conservation Biology Institute, National Zoological Park, Washington, DC 20013-7012 USA; 2 School of Natural Resources and Environment, University of Florida, Gainesville, FL 32611 USA; 3 Smithsonian-Mason School of Conservation, Smithsonian Conservation Biology Institute, National Zoological Park, Front Royal, VA 22630 USA

## Abstract

Linear infrastructure development and resulting habitat fragmentation are expanding in Neotropical forests, and arboreal mammals may be disproportionately impacted by these linear habitat clearings. Maintaining canopy connectivity through preservation of connecting branches (i.e. natural canopy bridges) may help mitigate that impact. Using camera traps, we evaluated crossing rates of a pipeline right-of-way in a control area with no bridges and in a test area where 13 bridges were left by the pipeline construction company. Monitoring all canopy crossing points for a year (7,102 canopy camera nights), we confirmed bridge use by 25 mammal species from 12 families. With bridge use beginning immediately after exposure and increasing over time, use rates were over two orders of magnitude higher than on the ground. We also found a positive relationship between a bridge’s use rate and the number of species that used it, suggesting well-used bridges benefit multiple species. Data suggest bridge use may be related to a combination of bridge branch connectivity, multiple connections, connectivity to adjacent forest, and foliage cover. Given the high use rate and minimal cost, we recommend all linear infrastructure projects in forests with arboreal mammal populations include canopy bridges.

## Introduction

Linear infrastructure, such as pipelines, roads, railways, and transmission lines, has the potential to impact wildlife in many ways, most notably reducing access to resources and increasing mortality^1–3^. These impacts can be even more pronounced in tropical forests, due to their extreme physical and ecological complexity^4^. In addition, many bird and small mammal species in tropical forests either avoid edges and/or are adverse to crossing clearings^5–7^, and arboreal species can be particularly reluctant to cross open clearings^8, 9^. For many species, crossing an open clearing on the ground represents a major predation threat, exemplified in a study by Campbell *et al*.^10^ by lower rates of terrestrial behaviour where predation pressure was higher. In the Neotropics, records of terrestrial behaviour in primates, one of the most well-studied groups of arboreal mammals, tend to be very rare^10, 11^, and they often involve cautious behaviours such as running^12^, displays associated with tension^13^, and use of the shortest path available to cross the clearing^14^.

Because of an aversion to using the ground, arboreal mammals may lose access to resources on the other side of a linear infrastructure clearing. However, expanding territory on one side of the clearing and searching for new resources risks infiltrating the territory of neighbouring individuals or groups, and encounters with those groups could be agonistic^15–17^. As with other stressors such as predation and social pressures, reduced resource access and increased conflict with neighbours may lead to decreased reproductive success, increased disease susceptibility, increased aggression and intra-group conflict, and a host of other challenges^18–20^.

Reducing the impacts of some forms of linear infrastructure like roads, transmission lines, and railways on arboreal wildlife is challenging for various reasons^4^. These forms of infrastructure represent a long-term source of forest fragmentation. They also tend to involve very wide forest clearings, preventing natural canopy connectivity. Finally, both roads and railways have continuous traffic, a source of animal-vehicle collisions. In contrast, buried pipelines may pose a threat that is more easily mitigated. Pipeline right-of-way (RoW) clearings can be as narrow as 4–15 m. Once pipelines are built, regrowth in the RoW may be allowed or even encouraged for erosion control. The combination of formal reforestation efforts and natural regeneration along well-managed pipelines allows these narrow clearings to recuperate, thereby reducing the length of time the forest is fragmented.

Where clearings are narrow, there is also higher potential for maintaining canopy connectivity during and after linear infrastructure construction. A sustained connection created by branches of trees on either side of an introduced linear feature, which provides a natural arboreal route from one side of the clearing to the other, is known as a canopy bridge. In Ecuador, attempts have been made to preserve some natural canopy connectivity over pipeline RoWs, with one project leaving the canopy nearly intact along the length of a RoW^21^ and another leaving 40 m-long canopy bridges every 1.8 km^22^. An additional project in Costa Rica documented the reestablishment of canopy connectivity over a road^23^. However, none of these studies quantified the frequency of use of bridges by arboreal mammals or the relative frequency of crossing on the ground versus in the canopy. Natural canopy bridges may represent a promising way to minimize the effects of forest fragmentation on arboreal animals. However, it is necessary to document the degree to which these bridges are used by various taxa and the extent to which these taxa can and will cross RoW clearings on the ground.

In this study, we tracked the use of natural canopy bridges and pipeline RoW clearings by arboreal wildlife with camera traps during and after the construction of a natural gas pipeline. The goals of our study were three-fold: 1) to confirm which, if any, arboreal mammal species used natural canopy bridges, 2) to assess overall bridge use frequency and factors that affect bridge use, including bridge characteristics, and, 3) to evaluate the overall utility of the bridges by comparing bridge crossing frequency to ground crossing frequency.

## Results

### Crossing Rates before Construction

Before construction, primate groups were observed crossing at the canopy level over the proposed RoW at a rate of 2.7 (25 crossings) and 3.6 (29 crossings) times/10 km walked in the Bridge Zone (BZ) and No Bridge Zone (NBZ), respectively. Of all groups observed during transect walks, 76% and 81% crossed in the BZ and NBZ, respectively. These data confirmed that primate groups crossed the RoW in both zones prior to its construction, suggesting that territories of those groups incorporated forest on both sides of the RoW.

### Monitoring Canopy Bridge Use

Over the year-long study, we logged a total of 7,102 camera nights in the canopy and 4,182 and 2,972 camera station nights on the ground in the BZ and NBZ, respectively. Due primarily to extremely humid conditions and damage by animals (e.g. ants, termites, and porcupines), the cameras experienced a high rate of malfunction. In the canopy, 1,072 potential trap nights were lost due to camera malfunctions, and on the ground, 1,346 and 4,103 trap nights were lost in the BZ and NBZ, respectively, due to malfunctions and vandalism. For ground cameras, station nights were still counted as long as one of the two cameras at a station was functioning properly.

**Figure 1:**
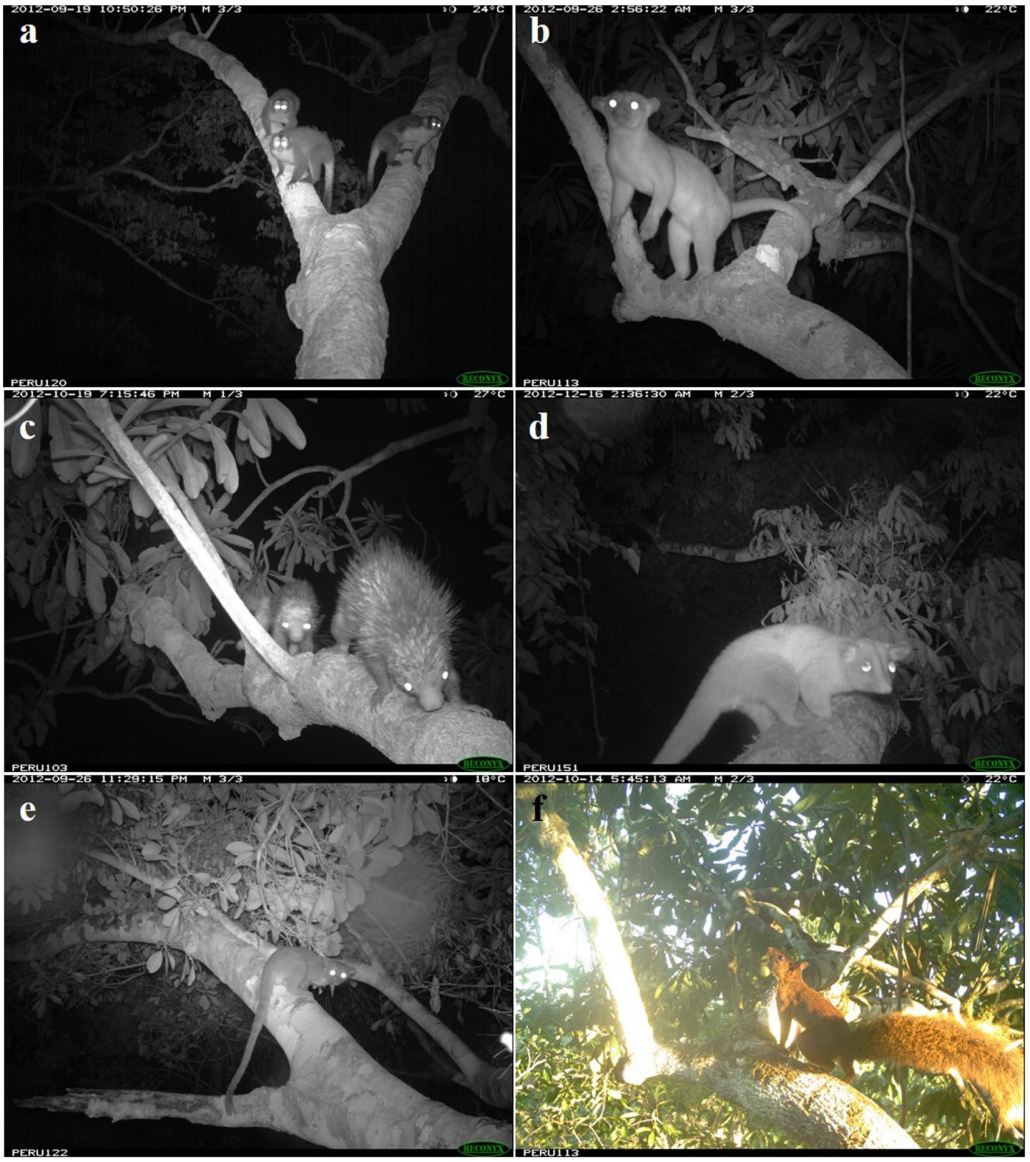
The six species that most frequently used the 13 natural canopy bridges over the pipeline clearing: (**a**) *Aotus nigriceps*, (**b**) *Potos flavus*, (**c**) *Coendou ichillus*, (**d**) *Caluromys lanatus*, (**e**) *Bassaricyon alleni*, and (**f**) *Hadrosciurus spadiceus*.

In the canopy, camera traps documented 25 species from 12 mammal families using the bridges in 3,372 photo events, 3,158 of which involved a bridge crossing (2,755) or a probable crossing (405), for an overall rate of 44.47 crossings per 100 trap nights (Table 1, Fig. 1). Using individually identifying markings and assuming different individuals based on group size and expected home range size, we conservatively estimated that a total of 150 individuals used the bridges. In all ground-level camera traps, we recorded 21 species belonging to 15 families (Supplementary Table 1); however, only six of these species overlapped with those recorded in the canopy. We recorded these six arboreal species in just six events in the BZ and 10 events in the NBZ, translating to 0.14 and 0.34 crossings per 100 station nights (0.22 overall on the ground), respectively (Table 1, Supplementary Fig. 1). In the canopy, 94.0% of crossings occurred at night, while on the ground 62.5% of the station events of arboreal species occurred at night (both zones combined).

**Table 1:** Arboreal and terrestrial mammal crossing events (and rates/100 camera trap/camera trap station nights) in the Bridge and No Bridge Zones.

Family	Species	Arboreal Crossing Events (rate/100 trap nights)		Terrestrial Crossing Events (rate/100 trap nights)			
		Nocturnal Events	Diurnal Events	Bridge Zone		No Bridge Zone	
				Nocturnal Events	Diurnal Events	Nocturnal Events	Diurnal Events
Aotidae	*Aotus nigriceps*	840 (11.83)	1 (0.01)				
Procyonidae	*Potos flavus*	613 (8.63)	2 (0.03)				
Didelphidae	*Caluromys lanatus*	556 (7.82)					
Erethizontidae	*Coendou ichillus*	345 (4.85)					
Procyonidae	*Bassaricyon alleni*	328 (4.61)					
Sciuridae	*Hadrosciurus spadiceus*	4 (0.05)	67 (0.94)		1 (0.02)	1 (0.03)	3 (0.10)
Cebidae	*Sapajus apella*	4 (0.05)	53 (0.75)				3 (0.10)
Echimyidae	*Mesomys hispidus*	44 (0.61)					
Cebidae	*Cebus albifrons*	6 (0.08)	25 (0.35)		2 (0.05)		
Didelphidae	*Didelphis marsupialis*	26 (0.36)		2 (0.05)		2 (0.07)	
Myrmecophagidae	*Tamandua tetradactyla*	17 (0.23)	1 (0.01)	1 (0.02)			
Erethizontidae	*Coendou bicolor*	15 (0.21)					
Sciuridae	*Notosciurus pucheranii*		13 (0.18)				
Pitheciidae	*Pithecia irrorata*		12 (0.17)				
Megalonychidae	*Choloepus hoffmanni*	11 (0.15)					
Cebidae	*Saguinus imperator*		9 (0.13)				
Pitheciidae	*Callicebus brunneus*		4 (0.06)				
Didelphidae	Didelphidae sp. 1	4 (0.05)					
Cebidae	*Saguinus fuscicollis*		3 (0.04)				1 (0.02)
Cricetidae	*Oecomys* sp.	2 (0.02)					
Didelphidae	Didelphidae sp. 2	2 (0.02)					
Atelidae	*Ateles chamek*		1 (0.01)				
Atelidae	*Lagothrix cana*		1 (0.01)				
Cricetidae	*Rhipidomys* cf. *gardneri*	1 (0.01)					
Didelphidae	*Marmosa (Micoureus) demerarae*	1 (0.01)					
Procyonidae	*Potos flavus* or *Bassaricyon alleni*	120 (1.68)					
Unknown		38 (0.53)	3 (0.04)				
Total events (overall rates)		2963 (41.72)	195 (2.76)	3 (0.07)	3 (0.07)	3 (0.10)	7 (0.24)
Total events (overall rates)		3158 (44.47)	6 (0.14)	10 (0.34)			

Nocturnal (18:00–5:59) and diurnal (6:00–17:59) events are also presented. The 14 events that included two species were counted for each species but only once in the total. In some events of *Potos flavus* and *Bassaricyon alleni* it was not possible to distinguish the species, and in some cases the species could not be identified at all and was labelled “unknown.”

**Figure 2:**
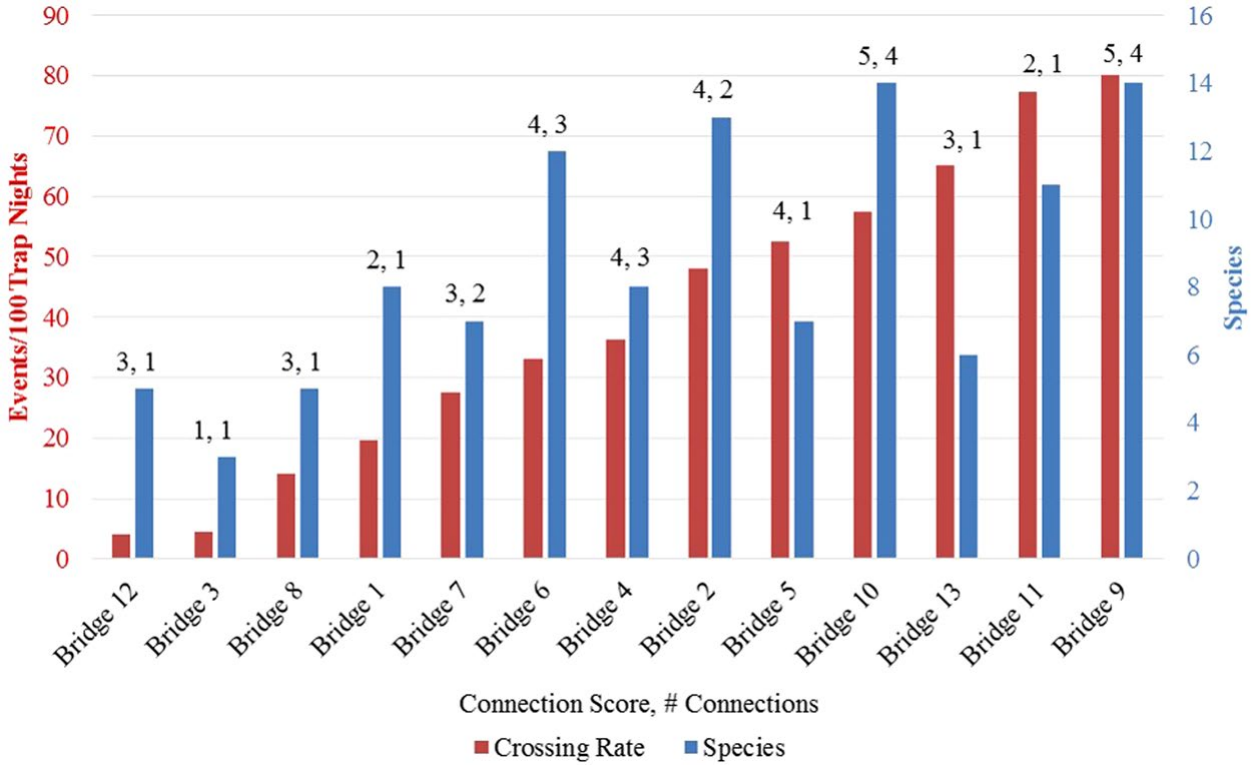
Overall crossing rate for all connections (events/100 trap nights) in each of the 13 natural canopy bridges over the natural gas pipeline clearing, and the total number of species registered in each bridge. The connection score (1–5,﻿ see text) and the number of connections (1﻿–4﻿)﻿ are indicated above each pair of columns.

Use of the 13 bridges varied widely (*M* = 39.99, *SD* = 25.82 crossings/100 trap nights, range: 4.55–80.08), as did connection use rates within each bridge, in cases where there were more than one (e.g. Bridge 9, four connections: *M* = 76.51, *SD* = 67.53 crossings/100 trap nights, range: 31.91–177.10, Fig. 2). The number of species that used each bridge also varied among bridges (*M* = 8.69, *SD* = 3.71 species per bridge, range: 3–14, Fig. 2), and there was a strong positive correlation between the rate at which a bridge was used and the number of species that used a bridge (*t*(12) = 4.81, *p* = 0.0002, *R* ^*2*^ = 0.63, Fig. 2). With regard to the analyses of bridge preference, none of the individual bridge characteristics we measured demonstrated a significant relationship with the crossing rate; although the analysis of the connection score (a subjective score assigned by TG and FCR that took into account various bridge qualities) approached significance (*p* = 0.09, Table 2).

**Table 2:** Results of regression analyses of crossing rates in relation to characteristics of the 13 bridges during the entire study period (Sept 2012–Sept 2013).

Bridge Characteristic	*F*	*R* ^*2*^	Slope	*p*
Mean DBH of bridge tree trunks	0.006	0.001	0.013	0.941
Distance between bridge tree trunks	1.636	0.129	−2.229	0.227
Height of crossing branches	0.283	0.025	−0.841	0.605
Number of connections	2.098	0.160	8.701	0.175
Distance to next nearest bridge	0.145	0.013	−0.020	0.711
Connection score	3.449	0.239	10.672	0.090

Bridge 11 was not included in the analysis of the diameter at breast height (DBH) of the bridge tree trunks because it was composed of a liana connecting two trees rather than actual branches of the trees.

**Figure 3:**
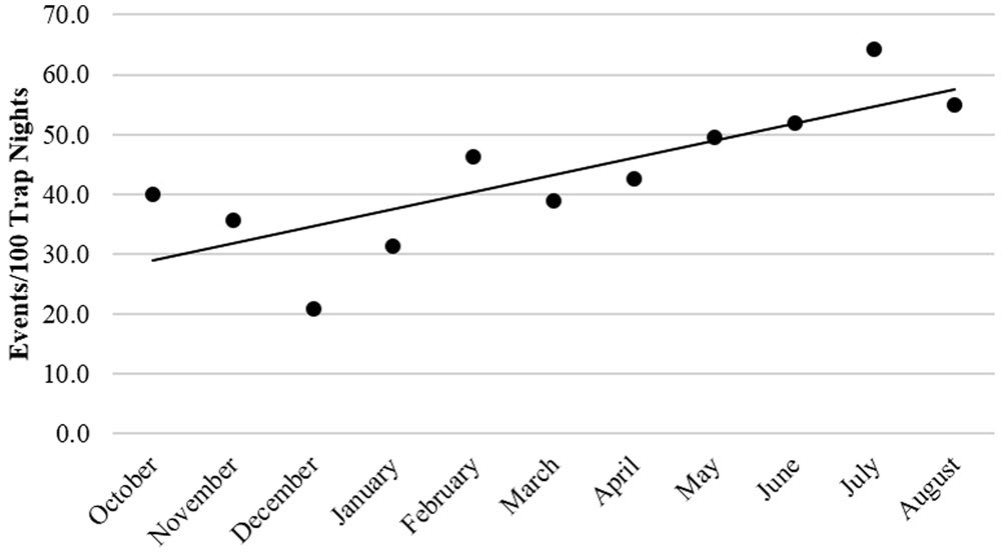
Events per 100 trap nights in all functioning connections of the 10 bridges that were still standing through August 2013 (September 2012 and 2013 were not included because not all camera traps were in place; *N* = 2,781 events; *R* ^*2*^ = 0.63, *p* < 0.05).

Finally, our analysis of monthly crossing rates in all of the bridges that remained standing through August 2013 (*N* = 10), demonstrated a steady increase in use over time (*R* ^*2*^ = 0.63, *F*(1,10) = 15.4, *p* = 0.002, Fig. 3).

## Discussion

Canopy bridges were used by a broad diversity of mammal species, with the six species that used the bridges the most representing five different families (Table 1 and Fig. 1). The 12 mammalian families we recorded during the year of sampling represent 86% (14 total) of those expected in the area based on the list published by Solari *et al*.^24^ for nearby Manu National Park (MNP). The two families found in MNP but not recorded in the bridges were Cyclopedidae (silky anteaters) and Bradypodidae (three-toed sloths). We also confirmed the presence of one unexpected species, *Coendou ichillus*, which we documented 900 km outside of its previously known range^25^.

Crossing rates of arboreal mammals were over two orders of magnitude higher in canopy bridges than on the ground. On the other hand, crossings on the ground demonstrate that the RoW did not entirely fragment the forest for all arboreal species. However, the fact that there were so few ground crossings (16 of 3174 total or 0.5%) and no ground crossing events by 19 of the 25 species documented in the bridges suggests that even linear infrastructure with no motorized traffic and little human use presents a substantial barrier to animal movement. Furthermore, the difference in crossing rates on the ground between the NBZ and the BZ (0.34 vs 0.14 crossings/100 trap nights, respectively) effectively translates into just one more crossing per 500 trap nights (or one per 38.5 nights with 13 trapping stations) in the absence of bridges. These results suggest that natural bridges serve as an effective mitigation tool, and that in their absence, the majority of arboreal mammal species are likely to be negatively impacted by resulting fragmentation.

Although bridge use increased over time, we also observed primates crossing at least one canopy bridge within a week of its exposure in July 2012 (before cameras were installed). We posit that natural canopy bridges provide a substrate and structure requiring minimal habituation by arboreal mammals, and therefore resulting in rapid impact mitigation. Similarly, if canopy bridge branches form parts of paths used before fragmentation or are near previously known paths, they may provide even more immediate impact mitigation, potentially preventing animals from suffering from short-term resource access loss. Further experimental research is encouraged to confirm these hypotheses.

The benefit of bridges with high levels of connectivity was dispersed across a range of species, demonstrated by the correlation between bridge use rates and the number of species represented. Nonetheless, differential bridge use suggests that bridge characteristics may influence probability and rate of use. None of the basic physical descriptors we recorded for bridges were related to crossing rates, at least in a univariate perspective. Potential interactions between factors like bridge height and width, for example, could not be investigated with our low samples. However, our analysis of the relationship between crossing rates and our descriptor of bridge connectivity approached significance (*p* = 0.09). This result, along with our experience with arboreal mammals and the observations of other researchers^22, 26^ indicates that bridges will be used more frequently and by more species when they are 1) connected at multiple points, potentially providing multiple crossing options, 2) have branches in full contact, making them available both to animals that do and do not jump between branches, 3) are well connected to the adjacent forest, causing them to have a viable destination or be part of or near a pre-existing path, and 4) provide foliage cover, possibly reducing exposure to predation.

Those considering implementing natural canopy bridges in tropical forest will be interested to determine the minimum recommended distance between bridges. One natural canopy bridge project in Ecuador left bridges as far as two kilometres apart^22^, and Finer *et al*.^27^ recommended one bridge every kilometre. When evaluating the distance between bridges, it is helpful to understand forest structure and to know which arboreal species are present in the area and the size of their ecological neighbourhoods^28^ (i.e. their use-area). Bissonette and Adair^29^ provide recommendations for using allometric scaling to calculate appropriate distances between crossing structures that cater to the needs of species that use the landscape in differing ways. In our study area, species such as *Aotus nigriceps*, the heaviest bridge user, have mean home ranges as small as 9.2 ha (or 303 × 303 m; *N* = 9, range: 7–14 ha)^17^. Being characterized by aggressive intergroup encounters^17^, this species is unlikely to range outside of its territory to locate a bridge for crossing. Our analysis of bridge use in relation to the distance to the next bridge further supports this point, as we did not find higher use of more isolated bridges, which we would expect if animals were converging at bridges. Given the fact that habitat and territory size requirements vary broadly among the arboreal mammal species present in the study area^30^, widely separated bridges could exclude the territories of some species altogether. We therefore recommend that linear infrastructure projects in tropical forests leave bridges 300 m apart or closer, and we would not expect higher bridge density to result in fewer crossings per bridge. The location and number of canopy bridges, while a question of significant interest during planning phases of these projects, will be strongly dependent on engineering and design restrictions associated with the topography, slope, and pipeline characteristics. Nonetheless, the bottom line is that the more bridges there are, the higher the chances animals will find ones that suit their needs.

Ninety-four percent of bridge crossings occurred at night in this study and only six percent of crossings were during the day. While there are no canopy camera trapping studies with which to directly compare this value, a study by Whitworth *et al*.^31^ in nearby MNP demonstrated higher camera trapping rates of many of the diurnal primate species, particularly the Atelines (*Ateles chamek* = 0.28, *Lagothrix cana* = 1.88, and *Alouatta sara* = 0.42 events per 100 trap nights). On transect walks, we also found encounter rates of diurnal primates to be much lower than those found at Manu^32, 33^, potentially due to hunting by members of the local Matsigenka communities. Areas with lower hunting pressure, or large populations of diurnal primates in general, might demonstrate even higher crossing rates both in the canopy and on the ground.

It is important to note, with regard to our comparison of bridge crossings versus ground crossings, that each canopy camera covered less area per camera than each ground camera; while ground cameras sampled a plane, canopy cameras sampled a series of branches (or, essentially, limited pathways). On the other hand, the proportion of the potential crossing area sampled was much lower for ground cameras than canopy cameras, which effectively sampled 100% of all canopy crossing points when all cameras were functioning. Indeed anecdotal observations suggest that there may have been more crossing on the ground than detected by the ground-level cameras. After observing a group of *Saguinus fuscicollis* crossing the RoW on the ground using a log that partially spanned the clearing, a camera trap was set up at that location. We recorded three more crossing events at this location, all by *Saguinus imperator*, in a span of 186 days. While cameras were set specifically to photograph all crossings in the canopy, differential detectability of different species (e.g. based on body size and behaviour^34^) on the ground is certainly an existing bias when comparing capture rates from these stations. Addressing these issues was outside of the scope of this study and does make interpretation of our ground vs. canopy rates challenging. However, our results demonstrate unequivocally that natural bridges were used on a frequent basis by a diversity of arboreal mammals to cross the pipeline clearing while the ground was not.

An alternative method to maintain canopy connectivity is the use of artificial bridges, which have been implemented successfully in a variety of contexts^23, 26, 35–44^. Both natural and artificial bridges have their benefits and shortcomings. Some researchers have found artificial bridges to involve relatively long habituation times and/or complex or expensive construction logistics^41, 43–45^, while others have found the opposite: rapid habituation and/or inexpensive construction^38–40, 42^. Other studies have found lower than expected use of artificial bridges by some species^26^ or a preference for natural substrates like bamboo^46^ or even natural bridges^23^. On the other hand, in this study we found one drawback of natural canopy bridges to be loss of connectivity if trees fall or branches break during harsh weather events or due to helicopter traffic. While failure of artificial bridges is certainly possible as well, they are more easily repaired than natural bridges. To safeguard natural bridges, some studies have implemented methods to protect roots^21, 22^, and we recommend that future studies investigate ways to preserve bridge trees and reduce the harmful effects of sudden exposure of the trunks and roots to both climatic elements such as sun and wind and anthropogenic factors such as vehicular traffic that can damage roots.

Results from this study and others suggest that the optimal bridge type is likely to be context specific. When choosing to use natural or artificial bridges or when selecting the design for artificial bridges, it is important to evaluate which type of bridge will be most effective in the given situation (e.g. linear infrastructure feature type, width of clearing, type of vegetation in the area, budget for bridges, long-term plan for use of the infrastructure) and for the arboreal species present in the area. Thus far, only one study has directly compared artificial and natural canopy bridge use^23^. Our understanding of arboreal mammal proclivity to use different bridges will benefit from more studies comparing bridge types and further research into a variety of artificial bridge designs. No matter which mitigation method is used, the sooner it is installed the better. Arboreal animals will immediately experience stress related to resource access loss when the canopy is fragmented.

Through discussions with construction company employees, we determined that the cost incurred by a company of leaving natural canopy bridges over a pipeline is almost non-existent given proper planning^47^. Canopy bridges only involve a cost if the trunks of the trees that form them are very close together (e.g. 8 m—Bridge 9, Supplementary Fig. 1). With prior planning, a path for a pipeline can be selected that passes through areas with larger trees, which tend to have a higher likelihood of connecting across a wider clearing. Also, we found that there are various construction constraints that make leaving canopy bridges problematic—i.e. tree trunks cannot easily be preserved on steep ridges or on sharp turns. Conditions for canopy bridges can be more favourable if these constraints are managed ahead of time. For example, planners can seek out paths in flatter areas or areas that require fewer turns.

Our results demonstrate that canopy bridges can be highly effective in fragmentation mitigation, particularly in the short term, and the results of this study can be used to support regulations recommending implementation of natural canopy bridges as a mitigation practice in linear infrastructure construction, particularly pipelines. Natural canopy bridges may also be effective over roads, as found by Lindshield^23^, and the maintenance of canopy connectivity should be considered in any linear infrastructure project where arboreal mammals are thought to be present, given that the benefits are now clearly demonstrated and costs can be minimal. Finally, given the current relative rarity of this practice, we strongly encourage all projects utilizing natural bridges to closely monitor and collect data regarding their use. Doing so will allow investigation of the factors most important in dictating differential animal use of bridges so that mitigation benefits of each bridge can be maximized.

## Methods

### Study site

**Figure 4:**
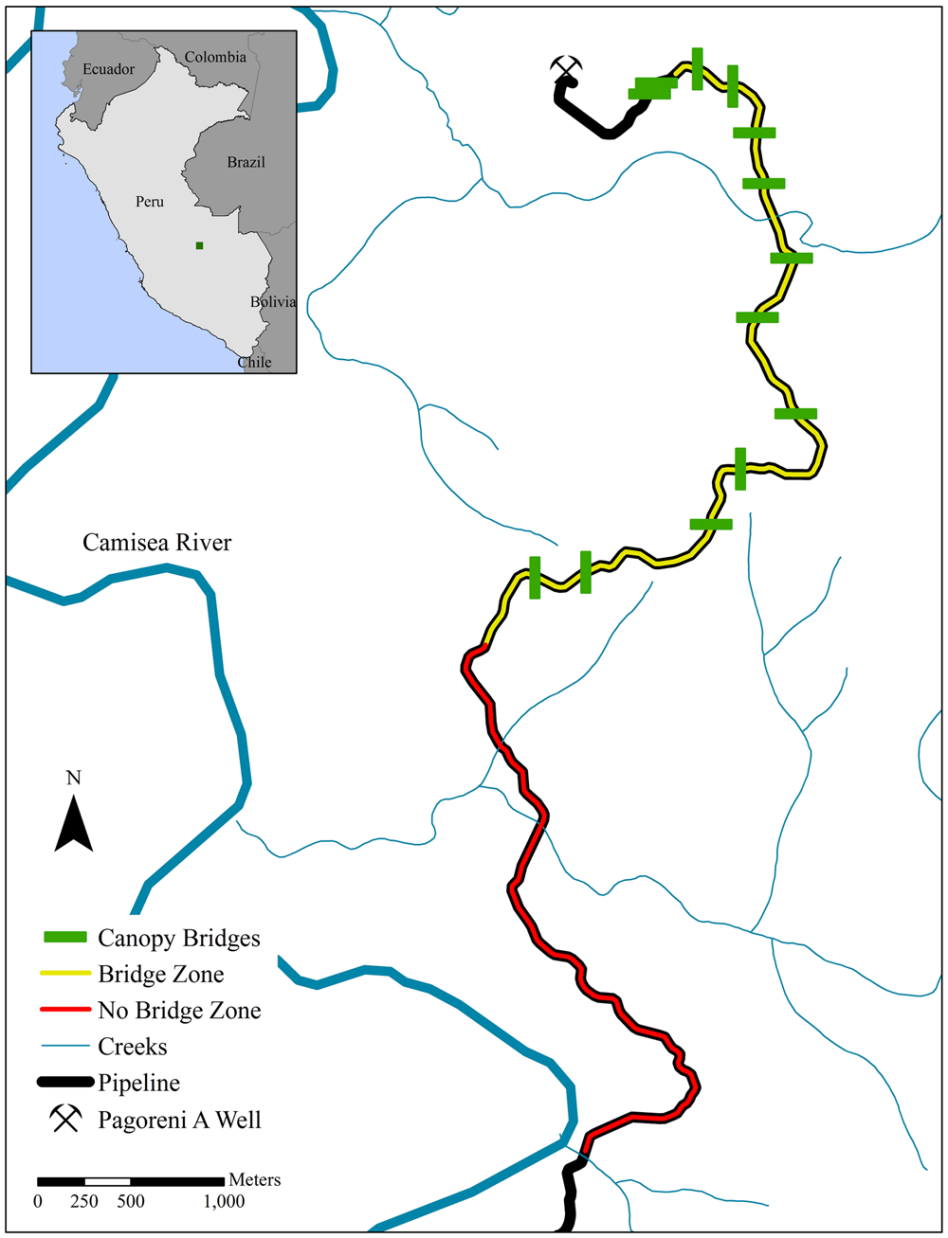
Map of the study area, including the location of natural canopy bridges (green bars), the Bridge Zone (yellow line, 5.2 km), and the No Bridge Zone (red line, 4.0 km) along the pipeline right-of-way (black outline). Map created by T.G. in ArcMap 10.3 (Environmental Systems Resource Institute, ArcMap 10.2 ESRI, Redlands California, www.esri.com).

The study site is located in the Lower Urubamba Region of Peru (11°42′S, 72°48′W, Fig. 4) in hydrocarbon concession blocks 56 and 58 and is in close proximity to the Pagoreni A natural gas well. The area is topographically variable *terra firme* primary forest, with a dry season from May to September, a wet season from October to April, and 3000–3500 mm of annual rainfall^48^.

In January 2010, a topography team from the company that ultimately constructed the pipeline mapped the tentative natural gas pipeline RoW route^49^. Before construction began, we identified a Bridge Zone (BZ), a 5.2 km section of the proposed RoW where natural bridges would be left, and a No Bridge Zone (NBZ), a 4 km control area where bridges would not be left, (Fig. 4).

### Bridge Selection and Creation

**Figure 5:**
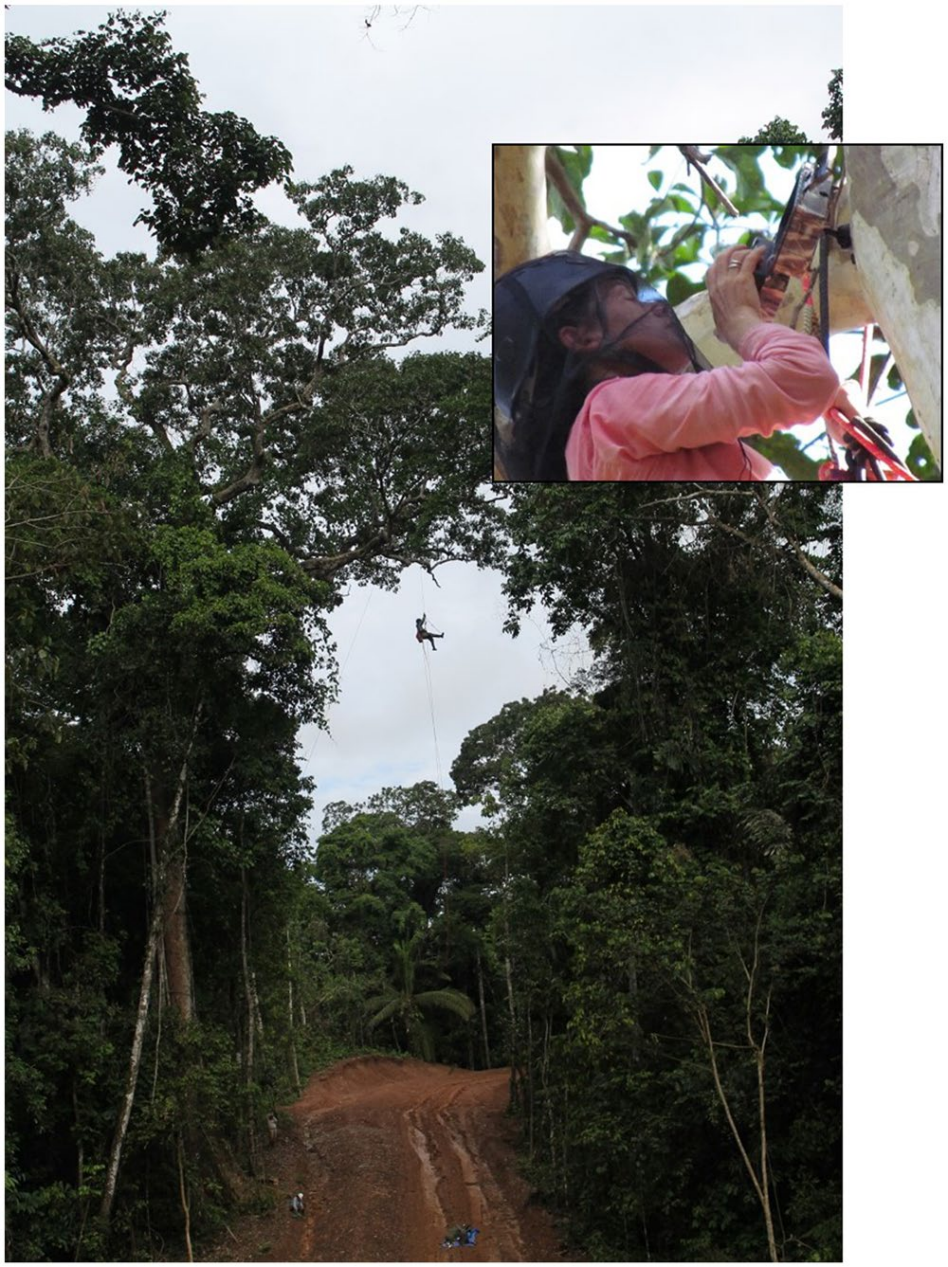
TG climbing a canopy bridge over a recently cleared natural gas pipeline in the Lower Urubamba Region of Peru to place and program (inset) camera traps.

Between March and November 2011, we evaluated the connectivity of the canopy above the proposed RoW, selecting 42 potential locations for future natural bridges in the BZ. Between April and July 2012, we accompanied the topography team mentioned above to ensure that the canopy bridge connections were preserved when possible during construction (see bridge selection criteria in ref. 47). The 10 to 25 m-wide RoW was cleared with excavators and the branches forming the canopy bridges were preserved between June and August 2012. After engineering constraints were considered and actual connectivity of each location was re-evaluated during construction, 13 canopy bridges were left, resulting in 25 connection points (some bridges included connections of more than one pair of branches). Eight of the bridges consisted of pairs of trees on opposite sides of the RoW, and the other five bridges were composed of connections between three or more trees (Fig. 5, Supplementary Fig. 1). Construction of the pipeline with heavy machinery continued until February 2013, and the daily presence of reforestation and inspection teams on foot continued until May 2013.

The trunk bases of the 13 bridges were an average of 14.7 m (*SD* = 4.2 m, range: 8.0–24.7 m, Supplementary Table 2) apart, and the bridges were an average of 415 m (*SD* = 225 m, range: 80–810 m, Supplementary Table 2) apart along the length of the RoW. Between September 2012 and September 2013, the canopy bridges suffered some damage, likely due to increased exposure and vulnerability of the tree trunks after pipeline clearing. After six months, 92% (23) of the original 25 bridge connections and all 13 bridges remained connected. By October 1, 2013, just over a year after the canopy cameras were placed, 52% of the connections (13) and 62% of the bridges (8) remained functional.

### Crossing Rates before Construction

In September–October 2011, before construction of the pipeline began, we evaluated crossing rates of primates in both the BZ and NBZ through census walks along the proposed pipeline route in both areas to verify that, with an intact canopy, crossing occurred in both zones. Only diurnal primates were included in this evaluation because their activity patterns and tendency to travel in relatively conspicuous social groups makes them easier to detect during the day than other arboreal mammals. Census walks occurred between 07:00 and 12:00 and between 13:00 and 17:00. Monitoring teams, composed of a local guide, a primatologist (TG or FCR), and a first-aid provider, walked at a rate of 1.0–1.2 km/hr., and both transects were monitored for a total of 93.6 km (18 times) and 80 km (20 times) in the BZ and NBZ, respectively.

When the research team observed a group of primates, they waited to see if the group crossed or did not cross from one side of the proposed RoW to the other. When the trajectory of the primate group seemed to be to cross but the monkeys turned back as an apparent reaction to the research team, the event was categorized as a “probable crossing.” Crossings and probable crossings were pooled for the evaluation.

### Monitoring RoW Crossing

In September 2012, after the construction process began and the bridges were exposed, we climbed the bridge trees and installed 25 Reconyx PC800 Hyperfire^TM^ Professional (Reconyx Inc., Holmen, WI, USA) camera traps in all possible crossing points (1–4 per bridge) at an average height of 26.8 m (range: 13.5–33.7 m) in each of the bridges (see ref. 50 for methods, Fig. 5). We carefully aimed the canopy cameras using a double ball joint mount^50^ to best capture animal crossings. We serviced the arboreal cameras during visits in October and December 2012, and in March 2013, replacing batteries, downloading photographs, and replacing malfunctioning cameras. Settings for arboreal cameras were as follows: three pictures per trigger, less than one second between photographs (“RapidFire”), no “quiet period” between triggers, and the image size was 3.1 MP. The cameras were equipped with 12 AA lithium batteries and 4 GB memory cards until early December 2012, when 16 GB cards were installed in all cameras.

In October 2012, we also installed 30 paired terrestrial camera traps below the bridges at ~50 cm above the ground in the BZ (2–3 per bridge depending on the length of the bridge area, i.e. some bridges included four trees and extended further along the RoW than others) and 26 more in pairs in the NBZ at 13 points at approximately 300 m intervals over the 4.0 km stretch. We serviced the cameras in December 2012 and March 2013. At each of these camera trapping stations, we installed one camera of each pair facing “down” the RoW (south-facing) and the other facing “up” the RoW (north-facing), placing the pairs of cameras on one tree on either the western or eastern side of the RoW, depending on the availability of large tree trunks. We used both Reconyx PC800 Hyperfire^TM^ and RC55 Rapidfire^TM^ camera traps.

The Passive Infrared Motion Detector in the Reconyx cameras has a field of sensitivity of 40° out to 30.5 m, depending on ambient conditions (Reconyx Hyperfire Instruction Manual, 2012). For this reason, with the RoW perpendicular to the space in between the cameras, we angled the cameras 40° apart from one another in opposite directions to maximize their coverage area (Supplementary Fig. 2). The PC800 cameras were equipped with 12 AA lithium batteries and 4 GB memory cards, and the RC55 cameras were equipped with six AA lithium batteries and 1 GB memory cards, changed to 4 GB cards in December 2012. Cameras in the BZ were distributed in such a way as to capture all RoW crossings that occurred under the bridges, accounting for the fact that bridges could be used by animals for crossings both on the ground and in the canopy. We removed all arboreal and ground camera traps in September 2013.

While we initially programmed ground cameras with the same settings as arboreal cameras, construction activities quickly resulted in thousands of irrelevant photos of crews and machinery. Therefore in December 2012, we reprogrammed ground cameras to maximize memory card and battery life (south-facing cameras: 10 photos per trigger, 1 second delay between triggers, and a one minute quiet period between triggers; north-facing cameras: 5 photos per trigger, 1 second delay, and 30 second quiet period). We programmed the north- and south-facing cameras differently to minimize the likelihood of both camera memory cards in a pair filling, rendering a camera station inoperative.

We tested all cameras *in situ* after each maintenance event to ensure functionality. For ground cameras, we performed a walk test to the opposite side of the RoW. For canopy cameras, we reviewed sample pictures once cameras were set up to ensure that the crossing point fell well within the camera’s sensitivity field.

For all arboreal photos, we evaluated whether an event qualified as a canopy bridge crossing, a probable crossing, or a non-crossing based on whether the animal travelled in front of the camera without backtracking (backtracking was very rare). Crossings and probable crossings were pooled for the analysis. Crossings by groups of animals were quantified as single events. Events of arboreal mammals on the ground were considered to be crossings of the RoW unless it was clear that the animal/s backtracked. Both on the ground and in the canopy, we considered photographs of the same species at the same camera (for the canopy) or camera trapping station (for the ground) separated by more than 1 minute as separate events.

In order to understand which bridge characteristics were associated with higher bridge use, along with data on the number of connections, the distance between the bridge tree trunks, and the distance between bridges, we also measured the diameter at breast height (DBH) of all trees forming each bridge and the height of the connection point with a range-finder. In addition, we developed a metric called the “connectivity score,” ranging from 1–5 (1 = a poorly connected bridge, 5 = a well-connected bridge). This score was given to each bridge in the field by TG and FCR at the onset of the study. This metric was a subjective measure that took into account the following attributes: the number of connecting points, the degree of connectivity of those points (i.e. branches in full contact, branch tips only in contact, or not in contact), the degree of connectivity of the bridge trees to the adjacent forest, and the degree of foliage cover from predators on the bridge branches (i.e. with or without foliage). For quality control of the connectivity score, TG and FCR discussed the characteristics of each bridge, carefully considering its accessibility and likely use by various arboreal mammal species.

We performed univariate linear regressions on bridge-use rates and all of the bridge attribute data to investigate which characteristics were related to use. We also performed a Pearson’s correlation to evaluate whether there was a relationship between the number of crossings in a bridge and the number of species to use that bridge. Finally, we evaluated bridge use over time, performing a regression analysis to test the null hypothesis that the number of events per month would remain constant over time.

## Electronic supplementary material

Supplementary info

## References

[1] Benítez-López A, Alkemade R, Verweij PA (2010). The impacts of roads and other infrastructure on mammal and bird populations: A meta-analysis. Biological Conservation.

[2] Jenkins AR, Smallie JJ, Diamond M (2010). Avian collisions with power lines: a global review of causes and mitigation with a South African perspective. Bird Conservation International.

[3] van der Ree, R., Gulle, N., Holland, K., Grift Evd Mata, C. & Suarez, F. Overcoming the Barrier Effect of Roads-How Effective Are Mitigation Strategies? (2007).

[4] Laurance WF, Goosem M, Laurance SGW (2009). Impacts of roads and linear clearings on tropical forests. Trends in Ecology and Evolution.

[5] Laurance WF (2002). Ecosystem decay of Amazonian forest fragments: A 22-year investigation. Conserv Biol.

[6] Goosem M (2001). Effects of tropical rainforest roads on small mammals: Inhibition of crossing movements. Wildl Res.

[7] Develey PF, Stouffer PC (2001). Effects of roads on movements by understory birds in mixed-species flocks in Central Amazonian Brazil. Conserv Biol.

[8] Wilson RF, Marsh H, Winter J (2007). Importance of canopy connectivity for home range and movements of the rainforest arboreal ringtail possum (Hemibelideus lemuroides). Wildlife Research.

[9] Soanes, K. & van der Ree, R. Reducing Road Impacts on Tree-Dwelling Animals. In: *Handbook of Road Ecology* (ed(eds). John Wiley & Sons, Ltd (2015).

[10] Campbell CJ (2005). Terrestrial behavir in *Ateles* spp. Int J Primatol.

[11] Barnett AA (2012). Terrestrial activity in Pitheciins (*Cacajao, Chiropotes*, and *Pithecia*). Am J Primatol.

[12] Bicca-Marques JC, Calegaro-Marques C (1995). Locomotion of black howlers in a habitat with discontinuous canopy. Folia Primatol.

[13] Dib LRT, Oliva AS, Strier KB (1997). Terrestrial travel in muriquis (Brachyteles arachnoides) across a forest clearing at the Estacao Biologica de Caratinga, Minas Gerais, Brazil. Neotropical Primates.

[14] Martinez J, Wallace RB (2011). First observations of terrestrial travel for Olalla’s titi monkey (*Callicebus olallae*). *Neotropical*. Primates.

[15] Shaffer CA (2013). Activity patterns, intergroup encounters, and male affiliation in free-ranging bearded sakis (*Chiropotes sagulatus*). International Journal of Primatology.

[16] Perry S (1996). Intergroup encounters in wild white-faced capuchins (*Cebus capucinus*). Int J Primatol.

[17] Wright PC (1989). The nocturnal primate niche in the New World. Journal of Human Evolution.

[18] Honess PE, Marin CM (2006). Behavioural and physiological aspects of stress and aggression in nonhuman primates. Neuroscience and Biobehavioral Reviews.

[19] de la Torre S, Snowdon CT, Bejarano M (2000). Effects of human activities on wild pygmy marmosets in the Ecuadorian Amazon. Biol Conserv.

[20] Cavigeli SA (1999). Behavioural patterns associated with faecal cortisol levels in free-ranging female ring-tailed lemurs. Lemur catta. Anim Behav.

[21] Williams B (1999). ARCO’s Villano project: Improvised solutions in Ecuador’s rainforest. Oil and Gas Journal.

[22] Thurber, M. & Ayarza, P. Canopy bridges along a rainforest pipeline in Ecuador. *Society of Petroleum Engineers* (2005).

[23] Lindshield, S. M. Protecting Nonhuman Primates in Peri-Urban Environments: A Case Study of Neotropical Monkeys, Corridor Ecology, and Coastal Economy in the Caribe Sur of Costa Rica. In: *Ethnoprimatology: Primate Conservation in the 21st Century* (ed(eds Waller MT). Springer International Publishing (2016).

[24] Solari S, Pacheco V, Luna L, Velazco PM, Patterson BD (2006). Mammals of the Manu Biosphere Reserve. Fieldiana Zoology, NS.

[25] Gregory T, Lunde D, Zamora Meza HT, Carrasco-Rueda F (2015). Records of *Coendou ichillus* from the Lower Urubamba Region of Peru. Zookeys.

[26] Donaldson, A. & Cunneyworth, P. Case Study: Canopy bridges for primate conservation. In: *Handbook of Road Ecology*. John Wiley & Sons, Ltd (2015).

[27] Finer M, Jenkins CN, Powers B (2013). Potential of best practice to reduce impacts from oil and gas projects in the Amazon. PLOS ONE.

[28] Addicott JF (1987). Ecological Neighborhoods: Scaling Environmental Patterns. Oikos.

[29] Bissonette JA, Adair W (2008). Restoring habitat permeability to roaded landscapes with isometrically-scaled wildlife crossings. Biological Conservation.

[30] Emmons, L. & Feer, F. *Neotropical Rainforest Mammals*: *A Field Guide*. The University of Chicago Press (1997).

[31] Whitworth A, Braunholtz LD, Huarcaya RP, MacLeod R, Beirne C (2016). Out on a Limb: Arboreal Camera Traps as an Emerging Methodology for Inventorying Elusive Rainforest Mammals. Tropical Conservation Science.

[32] Gregory, T., Carrasco-Rueda, F., Deichmann, J. L., Kolowski, J. & Alonso, A. Response of primates to natural gas pipeline construction in the Peruvian Amazon. *Biotropica*. (2017).

[33] Endo W (2010). Game Vertebrate Densities in Hunted and Nonhunted Forest Sites in Manu National Park, Peru. Biotropica.

[34] Rowcliffe MJ, Carbone C, Jansen PA, Kays R, Kranstauber B (2011). Quantifying the sensitivity of camera traps: An adapted distance sampling approach. Methods in Ecology and Evolution.

[35] Soanes K (2013). Movement re-established but not restored: Inferring the effectiveness of road-crossing mitigation for a gliding mammal by monitoring use. Biological Conservation.

[36] Teixeira ZF, Printes RC, Fagundes JCG, Alonso AC, Kindel A (2013). Canopy bridges as road overpasses for wildlife in urban fragmented landscapes. Biota Neotropica.

[37] Goldingay RL, Rohweder D, Taylor BD (2013). Will arboreal mammals use rope-bridges across a highway in Eastern Australia?. Aust Mammal.

[38] Weston N, Goosem M, Marsh H, Cohen M, Wilson R (2011). Using canopy bridges to link habitat for arboreal mammals: Successful trials in the Wet Tropics of Queensland. Aust Mammal.

[39] Valladares-Padua C, Cullen L, Padua S (1995). A pole bridge to avoid primate road kills. Neotropical Primates.

[40] Das, J., Biswas, J., Bhattacharjee, P. C. & Rao, S. S. Canopy bridges: An effective conservation tactic for supporting gibbon populations in forest fragments. In: *The Gibbons, Development in Primatology*: *Progress and Prospects* (eds Lappan, S., Whittaker, D. J.). Springer Science and Business Media (2009).

[41] Mass V (2011). Lemur bridges provide crossing structures over roads within a forested mining concession near Moramanga, Toamasina Province, Madagascar. Conservation Evidence.

[42] Yokochi K, Benici R (2015). A remarkably quick habituation and high use of a rope bridge by and endangered marsupial, the western ringtail possum. Nature Conservation.

[43] Thurber, M. W. & Abad, G. H. Rainforest connectivity strategies for oil and gas development. In: *Society of Petroluem Engineers* (2016).

[44] Lokschin LX, Rodrigo CP, Hallal Cabral JN, Buss G (2007). Power Lines and Howler Monkey Conservation in Porto Alegre, Rio Grande do Sul, Brazil. Neotropical Primates.

[45] Goosem, M., Weston, N. & Bushnell, S. Effectiveness of rope bridge arboreal overpasses and faunal underpasses in providing connectivity for rainforest fauna (2005).

[46] Narváez Rivera, G. M. & Lindshield, S. M. An experimental evaluation of crossing structures for New World monkeys in a Costa Rican wildlife sanctuary. In: *Joint meeting of the International Primatological Society and the American Society of Primatologists* (ed(eds) (2016).

[47] Gregory, T. *et al*. Methods to establish canopy bridges to increase natural connectivity in linear infrastructure development. *SPE Latin-America Conference on Health, Safety, Environment & Social Responsibility in the Oil and Gas Industry*, Jun 26–27 (2013).

[48] Alonso, A., Dallmeier, F., Campbell, P. & Nogueron, R. The Lower Urubamba Region, Peru. In: *Urubamba: The Biodiversity of a Peruvian Rainforest, SI/MAB Series #7* (eds Alonso, A., Dallmeier, F., Campbell, P.). Smithsonian Institution Press (2001).

[49] Walsh Peru, S. A. Estudio de Impacto Ambiental Proyecto de Desarrollo del Área Sur del Campo Kinteroni (2010).

[50] Gregory T, Carrasco Rueda F, Deichmann J, Kolowski J, Alonso A (2014). Arboreal camera trapping: taking a proven method to new heights. Methods in Ecology and Evolution.

