# Supplementary material for: Natural canopy bridges effectively mitigate tropical forest fragmentation for arboreal mammals
Source: Sci Rep. 2017 Jun 20;7:3892. doi: 10.1038/s41598-017-04112-x (PMC5478646; doi:10.1038/s41598-017-04112-x)
Supplement: Supplementary file 1 — Supplementary info [file 41598_2017_4112_MOESM1_ESM.doc]

**Natural canopy bridges effectively mitigate tropical forest fragmentation for arboreal mammals**

Tremaine Gregory, Farah Carrasco-Rueda, Alfonso Alonso, Joseph Kolowski, and Jessica L. Deichmann

**Supplementary Table 1.** Species recorded in the Lower Urubamba Region in ground-level camera trap stations under 13 canopy bridges (BZ) in a 5.2km pipeline section and in 13 locations in the no bridge zone (NBZ) in a 4km pipeline section.

| **Species** | **Family** | **Order** | **Common Name** | **BZ** | **NBZ** |
| --- | --- | --- | --- | --- | --- |
| *Mazama americana* | Cervidae | Artiodactyla | Red brocket deer | x | x |
| *Pecari tajacu* | Tayassuidae | Artiodactyla | Collared peccary | x | x |
| *Atelocynus microtis* | Canidae | Carnivora | Short-eared dog | x |  |
| *Leopardus pardalis* | Felidae | Carnivora | Ocelot | x | x |
| *Leopardus wiedii* | Felidae | Carnivora | Margay | x |  |
| *Panthera onca* | Felidae | Carnivora | Jaguar | x | x |
| *Puma concolor* | Felidae | Carnivora | Puma | x | x |
| *Eira barbara* | Mustelidae | Carnivora | Tyra | x | x |
| *Procyon cancrivorus* | Procyonidae | Carnivora | Crab-eating raccoon | x | x |
| *Dasypus* cf. *kappleri* | Dasypodidae | Cingulata | Greater long-nosed armadillo | x | x |
| *Priodontes maximus* | Dasypodidae | Cingulata | Giant armadillo | x | x |
| *Didelphis marsupialis* | Didelphidae | Didelphimorphia | Common opossum | x | x |
| *Sylvilagus brasiliensis* | Leporidae | Lagomorpha | Brazilian rabbit | x | x |
| *Tapirus terrestris* | Tapiridae | Perissodactyla | South American tapir | x | x |
| *Tamandua tetradactyla* | Myrmecophagidae | Pilosa | Southern tamandua | x |  |
| *Cebus albifrons* | Cebidae | Primates | White-fronted capuchin | x |  |
| *Saguinus fuscicollis* | Cebidae | Primates | Brown-mantled tamarin |  | x |
| *Sapajus apella* | Cebidae | Primates | Tufted capuchin |  | x |
| *Cuniculus paca* | Cuniculidae | Rodentia | Lowland paca | x | x |
| *Dasyprocta variegata* | Dasyproctidae | Rodentia | Brown agouti | x | x |
| *Hadrosciurus spadiceus* | Sciuridae | Rodentia | Southern Amazon red squirrel | x | x |

**Supplementary Table 2.** Characteristics and crossing rates of the 13 canopy bridges.

| **Bridge** | **Mean DBH (cm)*** | **Mean distance between trunks (m)**** | **Height of crossing point (m)** | **Connection score (5=excellent)** | **Number of Connections** | **Distance to nearest bridge (m)** | **Distance to next bridge (m)***** | **Overall crossing rate (events/100 trap nights)** |
| --- | --- | --- | --- | --- | --- | --- | --- | --- |
| 1 | 69.3 | 14.4 | 24 | 2 | 1 | 315 | 315 | 19.6 |
| 2 | 195.5 | 19.8 | 34 | 4 | 2 | 315 | 810 | 48.0 |
| 3 | 59.3 | 12.7 | 28 | 1 | 1 | 455 | 455 | 4.5 |
| 4 | 75.1 | 15.0 | 26 | 4 | 3 | 455 | 810 | 36.4 |
| 5 | 50.9 | 12.8 | 21 | 4 | 1 | 595 | 595 | 52.5 |
| 6 | 66.4 | 18.7 | 25 | 4 | 3 | 370 | 370 | 33.1 |
| 7 | 94.8 | 15.5 | 28 | 3 | 2 | 370 | 450 | 27.5 |
| 8 | 115.4 | 22.8 | 31 | 3 | 1 | 260 | 260 | 14.2 |
| 9 | 60.0 | 8.0 | 30 | 5 | 4 | 260 | 340 | 80.1 |
| 10 | 169.4 | 12.0 | 30 | 5 | 4 | 245 | 245 | 57.4 |
| 11 | NA | 10.0 | 15 | 2 | 1 | 245 | 245 | 77.3 |
| 12 | 112.5 | 12.0 | 25 | 3 | 1 | 80 | 80 | 4.0 |
| 13 | 65.6 | 18.0 | 30 | 3 | 1 | 80 |  | 65.1 |
| *Includes 2-4 bridge trees; Bridge 11 not included because it was a liana | | | | | | | | |
| **Includes distances between the 2-4 bridge tree trunks corresponding to a connection | | | | | | | | |
| ***Value not included in bridge use analysis | | | | | | | | |


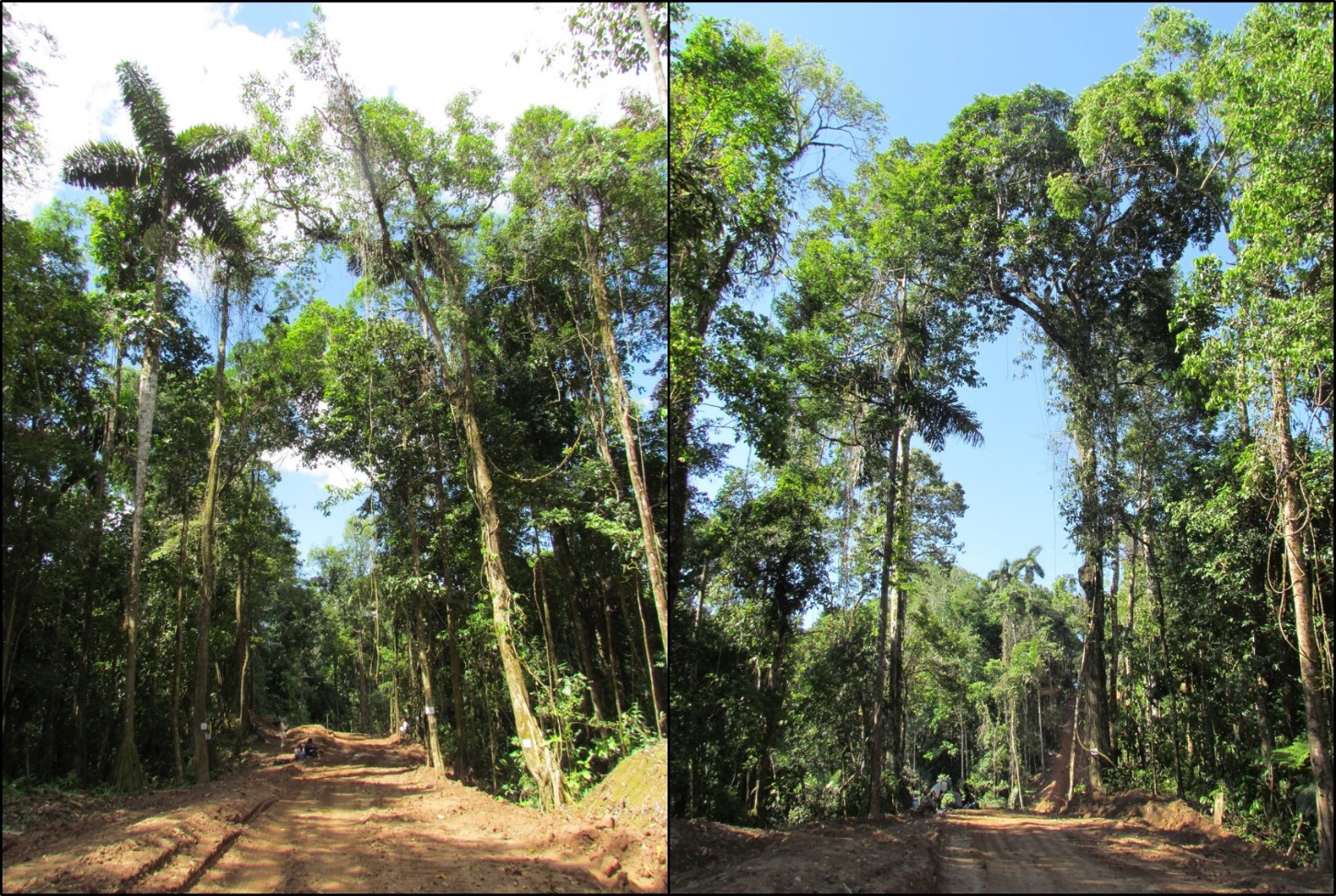


**Supplementary Figure 1.** Examples of canopy bridges (6, left, and 9) in October 2012 during pipeline construction. The RoW passes down the center of each photo, between the bridge trees.


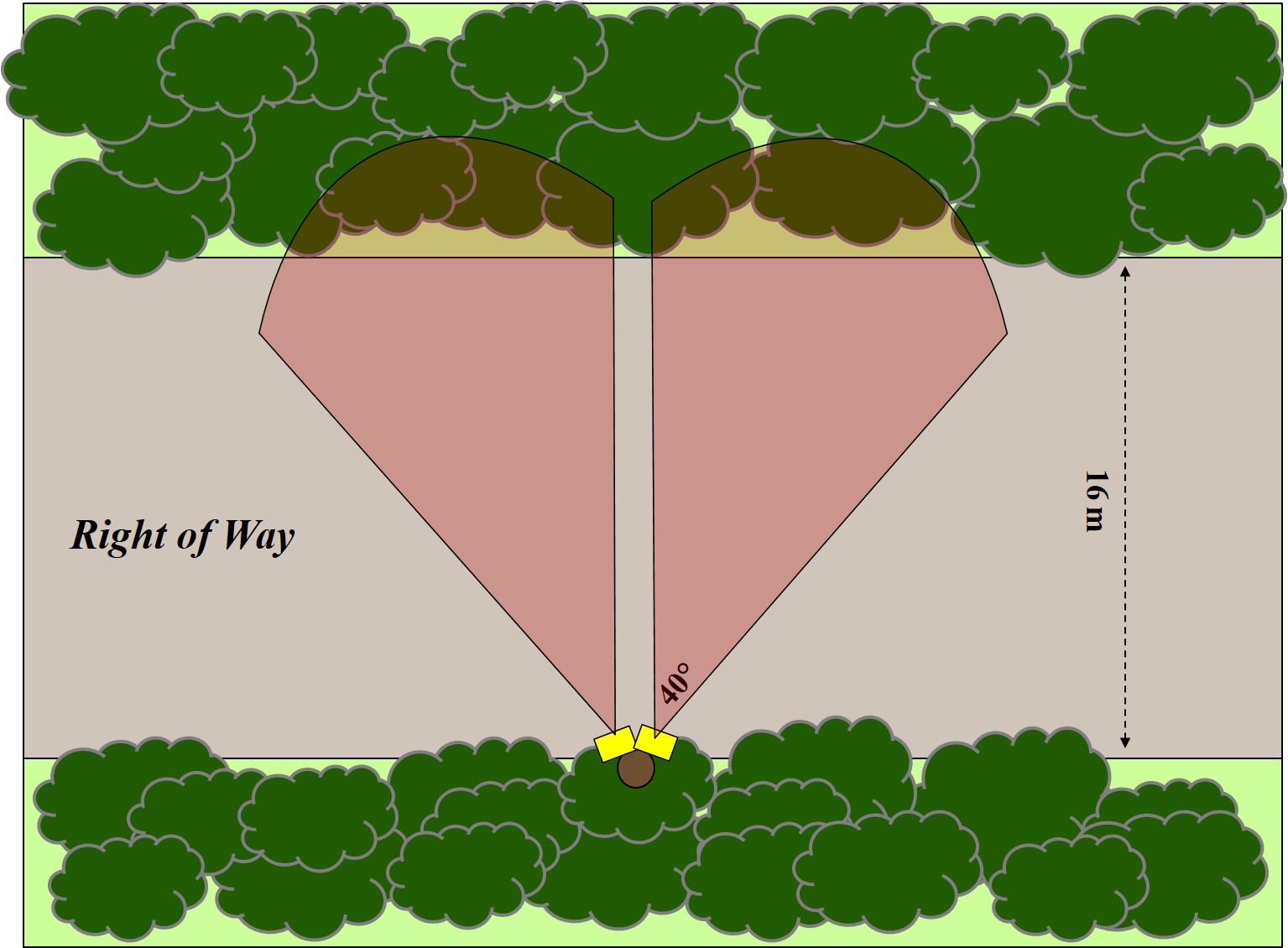


**Supplementary Figure 2.** Ground camera trap sampling scheme, including the RoW (light brown strip, here 16m in width), the paired cameras (yellow boxes), and their potential sampling areas (pink cones).


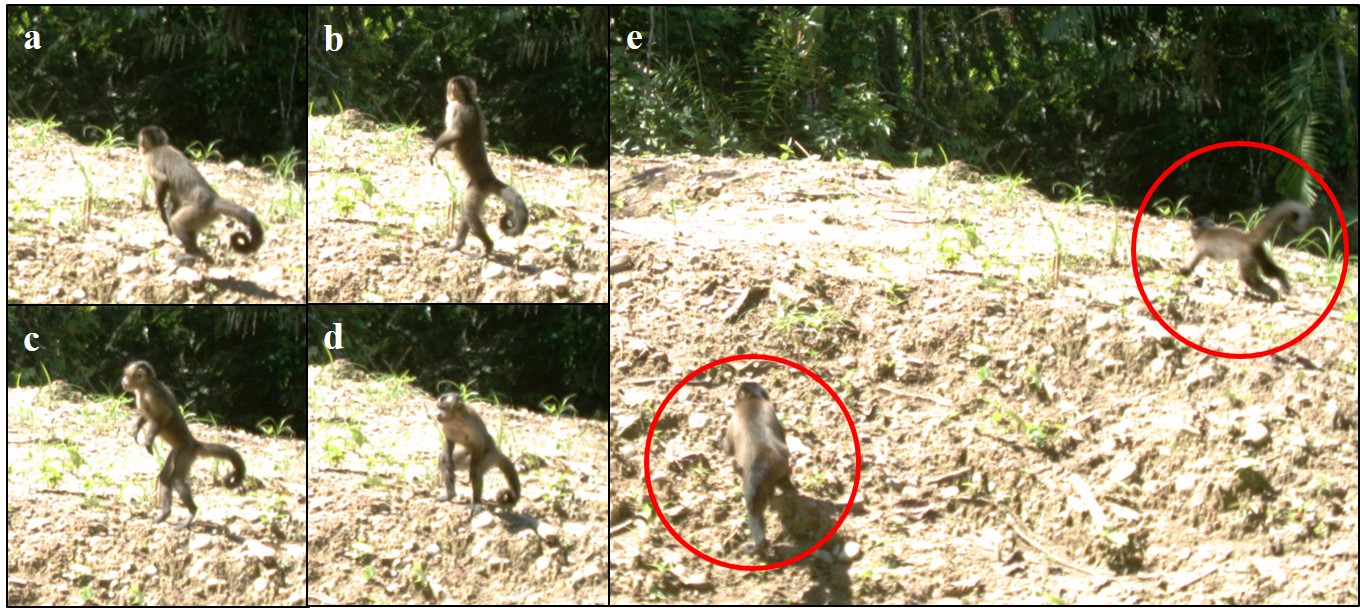


**Supplementary Figure 3.** Ground crossing event by a group of three capuchin monkeys, with one individual cautiously crossing first (a-d) and the other two individuals following quickly behind (e).
